# Clinical characteristics of hospitalized patients with false-negative severe acute respiratory coronavirus virus 2 (SARS-CoV-2) test results

**DOI:** 10.1017/ice.2021.146

**Published:** 2021-04-19

**Authors:** Erica L. MacKenzie, Dariusz A. Hareza, Maggie W. Collison, Anna E. Czapar, Antigone K. Kraft, Bennett J. Waxse, Eleanor E. Friedman, Jessica P. Ridgway

**Affiliations:** 1 Section of Infectious Diseases & Global Health, Department of Medicine, The University of Chicago Medicine, Chicago, Illinois; 2 Section of Internal Medicine, Department of Medicine, The University of Chicago Medicine, Chicago, Illinois; 3 Department of Pediatrics, The University of Chicago Medicine, Chicago, Illinois

## Abstract

**Objective:**

To determine clinical characteristics associated with false-negative severe acute respiratory coronavirus virus 2 (SARS-CoV-2) test results to help inform coronavirus disease 2019 (COVID-19) testing practices in the inpatient setting.

**Design:**

A retrospective observational cohort study.

**Setting:**

Tertiary-care facility.

**Patients:**

All patients 2 years of age and older tested for SARS-CoV-2 between March 14, 2020, and April 30, 2020, who had at least 2 SARS-CoV-2 reverse-transcriptase polymerase chain reaction tests within 7 days.

**Methods:**

The primary outcome measure was a false-negative testing episode, which we defined as an initial negative test followed by a positive test within the subsequent 7 days. Data collected included symptoms, demographics, comorbidities, vital signs, labs, and imaging studies. Logistic regression was used to model associations between clinical variables and false-negative SARS-CoV-2 test results.

**Results:**

Of the 1,009 SARS-CoV-2 test results included in the analysis, 4.0% were false-negative results. In multivariable regression analysis, compared with true-negative test results, false-negative test results were associated with anosmia or ageusia (adjusted odds ratio [aOR], 8.4; 95% confidence interval [CI], 1.4–50.5; *P* = .02), having had a COVID-19–positive contact (aOR, 10.5; 95% CI, 4.3–25.4; *P* < .0001), and having an elevated lactate dehydrogenase level (aOR, 3.3; 95% CI, 1.2–9.3; *P* = .03). Demographics, symptom duration, other laboratory values, and abnormal chest imaging were not significantly associated with false-negative test results in our multivariable analysis.

**Conclusions:**

Clinical features can help predict which patients are more likely to have false-negative SARS-CoV-2 test results.

Infection control practices to combat the spread of coronavirus disease 2019 (COVID-19) rely on rapid and accurate severe acute respiratory coronavirus virus 2 (SARS-CoV-2) testing. A wide variety of reverse-transcriptase polymerase chain reaction (RT-PCR) tests are available in the United States, although the sensitivity of these assays is variable.^1,2^ Of particular concern are false negative SARS-CoV-2 test results, which may occur because of low viral shedding, insufficient sample collection, incorrect handling, or variability in specimen site selection.^3,4^ False-negative rates have been reported to be between 2% and 54% in various studies.^5-8^ False-negative test results have important implications for hospital infection control because patients incorrectly identified as negative may be prematurely liberated from COVID-19 isolation precautions, resulting in risk of infection to other patients and staff.^9^ The Infectious Diseases Society of America (IDSA) currently recommends repeat testing 24–48 hours after an initial negative test result in patients with an intermediate or high clinical suspicion for COVID-19.^10^ However, determination of clinical suspicion may be challenging and may vary among healthcare settings. In addition, 2-step testing protocols result in increased use of isolation rooms, personal protective equipment, and other resources that may be in short supply.

Many studies have examined clinical variables associated with SARS-CoV-2 positivity in settings where RT-PCR testing is not available, but it is not clear whether these same clinical features predict COVID-19 among the subset of patients who initially test negative. To our knowledge, no study has examined whether integrating a complement of clinical variables, including comorbidities, laboratory results, and imaging findings, might be useful in differentiating true-negative test results from false-negative results in patients with potential COVID-19.^7,11–13^

## Methods

### Study design, definitions, and patient population

This retrospective observational cohort study included all patients 2 years of age and older tested for SARS-CoV-2 using RT-PCR between March 14, 2020, and April 30, 2020 at the University of Chicago Medicine, a tertiary-care facility on the south side of Chicago. Patients were included in the analysis if they met criteria for a COVID-19 testing episode, which we defined as any patient who had an initial SARS-CoV-2 RT-PCR test and then went on to have 1 or more additional tests within the following 7 days. The primary outcome measure was a false-negative testing episode, which we defined as an initial negative test followed by 1 or more positive tests within the subsequent 7 days. We defined a testing episode as a true negative if the initial test and all subsequent tests within 7 days were negative. Testing episodes in which the first test was positive were excluded from the analysis. If a patient had multiple testing episodes during the study period, only the first episode was included in the analysis.

During this period, hospital protocol dictated that testing be performed on all inpatients with any symptoms of influenza-like illness (eg, fever, chills, congestion, sore throat, cough, dyspnea, myalgias, nausea/vomiting, diarrhea) and outpatients with symptoms and significant risk factors (eg, high-risk chronic medical conditions, immunosuppression) or exposures (eg, known COVID-19–positive contacts or healthcare workers). Testing was repeated 48 hours later for all inpatients unless a clear alternative diagnosis was determined by an infectious disease physician. A diagnosis of COVID-19 was based on a positive SARS-CoV-2 test via nasopharyngeal swab using the Roche Cobas SARS-CoV-2 RT-PCR high-throughput assay (Roche Diagnostics, Basel, Switzerland) or the Cepheid Xpert Xpress SARS-CoV-2 RT-PCR assay (Cepheid, Sunnydale, CA) and the presence of symptoms. These assays have slightly different limits of detection (100 copies/mL for the Roche Cobas and 250 copies/mL for the Cepheid Xpert Xpress), but they show excellent agreement, even for specimens with low viral loads.^14,15^ The specific RT-PCR test used was included as a variable in the analysis to account for any potential difference in test sensitivity.

### Data collection

Information was collected from an electronic database of patients tested for COVID-19 including patient demographics, comorbidities, medications, vital signs, and the results of laboratory and radiographic studies. Presenting symptoms and risk factors were determined by chart review of notes in the electronic medical record. Vital signs were reviewed for the presence of fever (temperature >38°C) or hypoxemia (oxygen saturation <88% on 2 measurements or any use of supplemental oxygen or other respiratory support). Laboratory data were collected for test results previously shown to be associated with COVID-19, including C-reactive protein, ferritin, D-dimer, lactate dehydrogenase (LDH), liver function tests (aspartate transaminase and alanine transaminase), total white blood cell count, and lymphocyte count.^16–19^ Radiographic data, including the results of chest x-rays and computed tomography (CT) scans, were reviewed for common patterns including those known to be associated with COVID-19.^20,21^ Data on vital signs, laboratory results, and radiographic studies were included in the analysis if they were obtained within 24 hours of the first SARS-CoV-2 test.

### Statistical analysis

Baseline characteristics were evaluated using descriptive statistics. Discrete data are reported as frequencies and percentages. Continuous data are reported as medians and interquartile ranges. Significance testing was conducted using χ^2^ or Fisher exact tests as necessary for categorical variables and Wilcoxon rank-sum 2-sample test for continuous variables. Logistic regression was used to model associations between false-negative SARS-CoV-2 test results and demographics, laboratory results, clinical signs and symptoms, and risk factors. A multivariable model was created using variables with a *P* value < .05 from significance testing. For modeling, continuous variables were dichotomized at the median into high and low categories, and some race and ethnicity information was collapsed. Models examined the presence of a sign, symptom, or laboratory result against the absence or unknown status of that sign, symptom, or laboratory result. Odds ratios (ORs) and adjusted odds ratios (aORs) with accompanying 95% confidence intervals (95% CIs) are presented. All data analyses were conducted using SAS version 9.4 software (SAS Institute, Cary, NC).

### Ethics

This project underwent a formal review and was determined to be quality improvement, not human subjects research; therefore, it was not overseen by the institutional review board according to institutional policy.

## Results

**Fig. 1.**
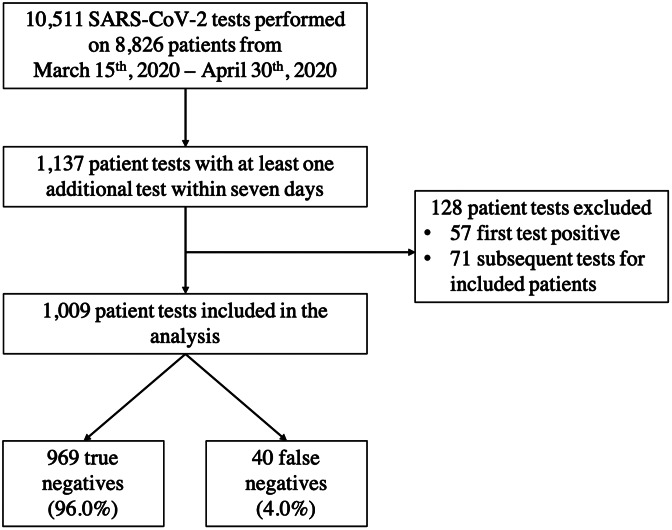
Flow diagram of SARS-CoV-2 tests included in the analysis. We evaluated all patients who underwent SARS-CoV-2 testing from March 15 through April 30, 2020. Patients were included in the analysis if they had at least 2 SARS-CoV-2 reverse-transcriptase polymerase chain reaction tests within 7 days. Patients were excluded if their first test was positive. If a patient had multiple testing episodes during the study period, only the first episode was included in the analysis.

In total, 10,511 SARS-CoV-2 tests were performed on 8,826 patients during the study period (Fig. 1). Of these, 1,137 tests were episodes in which an initial test was followed by 1 or more tests within 7 days. Moreover, 57 testing episodes were excluded from the analysis because the first test was positive, and an additional 71 were excluded because they were subsequent episodes for an already included patient. Of the remaining 1,009 testing episodes, 969 (96.0%) were true-negative results and 40 (4.0%) were false-negative results.

The baseline characteristics of the study population are shown in Table 1. The median age was 61 years, and 47.9% of patients were male; these factors did not differ significantly between patients with true-negative test results and false-negative test results. Most patients in the study were black non-Hispanic (69.1%). Patients with false-negative test results were less likely to be white non-Hispanic (18.4% vs 5.0%) and more likely to be Hispanic (10.0% vs 3.5%; *P* = .02 for all categories). The most common comorbidities for all patients included hypertension (70.6%), pulmonary disease (44.9%), congestive heart failure (33.8%), obesity (33.1%), chronic kidney disease (31.8%), and diabetes (31.5%). Patients with false-negative test results were less likely to have diabetes (16.7% vs 32.0%; *P* = .05) and cancer (8.3% vs 23.1%; *P* = .04), but they were similar to true-negative patients with regard to other comorbid illnesses. Almost all patients (96.2%) were inpatients at the time of initial testing and had repeated testing 48 hours later (48 hours for true-negative results vs 49 hours for false-negative results; *P* = .21).

**Table 1.** Baseline Characteristics and Testing Features of the Study Population

Characteristics	All Episodes	True Negative	False Negative	*P* Value
**Demographics**	n=1,009	n=969 (96.0%)	n=40 (4.0%)	
Age, median y [IQR]	61 [47–72]	61 [46–72]	61 [52–74.5]	.41^a^
Sex, male, no. (%)	483 (47.9)	462 (47.7)	21 (52.5)	.55
**Race/Ethnicity, no. (%)**				
Black Non-Hispanic	697 (69.1)	669 (69.3)	28 (70.0)	.02
White Non-Hispanic	180 (17.8)	178 (18.4)	2 (5.0)	
Hispanic	38 (3.8)	34 (3.5)	4 (10.0)	
Asian/Pacific Islander	16 (1.6)	16 (1.7)	0 (0.0)	
Other/multiple/unknown	78 (7.7)	72 (7.4)	6 (15.0)	
**Comorbidities, no. (%)**	n=957	n=921 (96.2%)	n=36 (3.8%)	
Diabetes	301 (31.5)	295 (32.0)	6 (16.7)	.05
Hypertension	676 (70.6)	654 (71.0)	22 (61.1)	.20
Obesity	317 (33.1)	302 (32.8)	15 (41.7)	.27
Pulmonary disease	429 (44.9)	412 (44.8)	17 (47.2)	.77
Coronary artery disease	233 (24.4)	224 (24.3)	9 (25.0)	.93
Congestive heart failure	318 (33.8)	310 (33.7)	8 (22.2)	.15
Chronic kidney disease	304 (31.8)	297 (32.3)	7 (19.4)	.11
Cancer	216 (22.6)	213 (23.1)	3 (8.3)	.04^b^
Cerebrovascular disease	103 (10.8)	100 (10.9)	3 (8.3)	.79^b^
Hyperlipidemia	295 (30.8)	287 (31.2)	8 (22.2)	.26
Venous thromboembolism	182 (19.0)	176 (19.1)	6 (16.7)	.71
Use of immunosuppressive medications (n=828), no. (%)	88 (10.6)	87 (10.8)	1 (4.4)	.50^b^
**Testing features**				
Inpatient at time of first test (n=1,009), no. (%)	971 (96.2)	932 (96.0)	39 (97.5)	.99^b^
First test Cepheid (n=894), no. (%)	401 (44.9)	386 (44.7)	15 (50.0)	.56
Time between tests (n=1,009), median h [IQR]	48 [47–51]	48 [47–51]	49 [48–82]	.21^a^

Note. IQR, interquartile range.

a Wilcoxon rank-sum 2-sample test.

b Fisher exact test.

Table 2 shows the presenting clinical features, laboratory values, and radiographic findings. The most commonly reported symptoms included dyspnea (42.0% of all patients), cough (31.9%), and fever (23.0%). Patients with false-negative test results were more likely to report anosmia or ageusia, although this was not a commonly reported symptom overall (7.7% vs 0.8%; *P* = .03). Patients with false-negative test results reported a longer duration of symptoms at the time of initial testing (7 days vs 3 days; *P* = .014). Having a known COVID-19–positive contact was associated with a significantly increased risk of false-negative testing (58.8% vs 11.3%; *P* < .0001), but there were no significant differences between false-negative and true-negative results among patients who were nursing-home residents or healthcare workers.

**Table 2.** Clinical Features, Laboratory Values, and Radiographic Results on Presentation

Characteristics	All Episodes	True Negative	False Negative	*P* Value
**Symptoms, no (%)**	*n=857*	*831 (97.0%)*	*26 (3.0%)*	
Subjective fever	197 (23.0)	190 (22.9)	7 (26.9)	.62
Chills	83 (9.7)	80 (9.6)	3 (11.5)	.73^a^
Congestion	56 (6.5)	53 (6.2)	3 (11.5)	.24^a^
Anosmia/ageusia	9 (1.1)	7 (0.8)	2 (7.7)	.03^a^
Cough	273 (31.9)	263 (31.7)	10 (38.5)	.46
Dyspnea	360 (42.0)	348 (41.9)	12 (46.2)	.66
Chest pain	120 (14.0)	118 (14.2)	2 (7.7)	.56^a^
Myalgias	60 (7.0)	56 (6.7)	4 (15.4)	.08^a^
Abdominal pain	132 (15.4)	132 (15.4)	4 (15.4)	.99^a^
Nausea/vomiting	153 (17.9)	150 (18.1)	3 (11.5)	.60^a^
Diarrhea	98 (11.4)	94 (11.3)	4 (15.4)	.53^a^
**Symptom duration (n=682), median d [IQR]**	3 [1–7]	3 [1–7]	7 [3–10]	.014^b^
**Risk factors, no. (%)**				
Resident of nursing home or other institution (n=693)	53 (7.7)	52 (7.7)	1 (4.8)	.99^a^
Healthcare worker (n=249)	15 (6.0)	14 (5.9)	1 (10.0)	.47^a^
Known COVID-19 contacts (n=292)	41 (14.0)	31 (11.3)	10 (58.8)	<.0001^a^
**Vital signs, no. (%)**				
Fever (n=897)	178 (19.8)	173 (19.9)	5 (17.9)	.99^a^
Hypoxemia (n=895)	357 (39.9)	339 (39.1)	18 (64.3)	.007
**Laboratory results, median [IQR]**				
White blood cell count, 10^3^/µL (n=874)	8.7 [6.0–12.7]	8.7 [6.0–12.8]	6.9 [5.6–9.0]	.03^b^
Lymphocyte count, 10^3^/µL (n=750)	1.3 [0.8–2.1]	1.3 [0.8–2.1]	1.1 [0.8–1.7]	.34^b^
Lactate dehydrogenase, U/L (n=181)	291 [220–423]	280 [215–398]	557 [291–609]	.008^b^
Aspartate transaminase, U/L (n=663)	29 [19–47]	29 [19–47]	37 [25–57]	.15^b^
Alanine transaminase, U/L (n=674)	21 [14–37]	21 [14–38]	25 [17–35]	.28^b^
C-reactive protein, mg/L (n=203)	49 [11–110]	48 [10–105]	68 [37–159]	.08^b^
D-dimer, µg/mL (n=130)	1.6 [0.9–4.2]	1.5 [0.9–3.5]	6.8 [1.5–16.8]	.05^b^
Ferritin, ng/mL (n=227)	356 [91–1,201]	319 [88–1,189]	938 [882–1,340]	.03^b^
**Microbiology results, no. (%)**	n=796	n=773 (97.1%)	n=23 (2.9%)	
Viral panel positive for another virus	64 (8.0)	64 (8.3)	0 (0.0)	.25
**Radiographic results, no. (%)**	n=735	n=712 (96.9%)	n=23 (3.1%)	
Abnormal pulmonary findings on chest imaging	464 (63.1)	444 (62.4)	20 (87.0)	.02^a^
**Predominant radiographic pattern, no. (%)**				
Airspace filling	35 (4.8)	31 (4.4)	4 (17.4)	.02^a^
Atelectasis	57 (7.8)	56 (7.9)	1 (4.4)	.99^a^
Focal opacity/pneumonia	23 (3.1)	23 (3.2)	0 (0.0)	.99^a^
Interstitial markings	114 (15.5)	110 (15.5)	4 (17.4)	.80^a^
Nodule(s) or mass(es)	21 (2.9)	21 (2.8)	1 (4.4)	.49^a^
Pleural effusion	27 (3.7)	27 (3.8)	0 (0.0)	.99^a^
Nonspecific opacities	187 (25.4)	177 (24.9)	10 (43.5)	.04^a^

Note. IQR, interquartile range.

a Fisher exact test.

b Wilcoxon rank-sum 2-sample test.

On presentation, patients with false-negative test results were more likely to be hypoxemic (64.3% vs 39.1%; *P* = .007) but not febrile (17.9% vs 19.9%; *P* = .99). Patients with false-negative test results had lower total white blood cell counts (6.9 vs 8.7 10^3^/µL, p = 0.03), higher LDH levels (557 vs 280 U/L; *P* = .008), higher D-dimer values (6.8 vs 1.5 µg/mL; *P* = .05), and higher ferritin levels (938 vs 319 ng/mL; *P* = .03) compared to patients with true-negative results. We detected no significant differences with regard to other laboratory values, including lymphocyte count, liver function tests, and C-reactive protein. Viral panel testing identified alternate viral pathogens in 8.3% of true-negative test results but in no false-negative test results, although this difference was not statistically significant (*P* = .25). Patients with false-negative testing were more likely to have abnormal pulmonary findings on chest imaging (87.0% vs 62.4%; *P* = .02). The most common radiographic pattern identified for patients with false-negative results was nonspecific opacities (43.5% among false-negative results) followed by interstitial markings and airspace filling opacities (17.4% each). Airspace filling opacities were significantly more common in patients with false-negative test results than in patients with true-negative test results (17.4% vs 4.4%; *P* = .02).

Table 3 displays the results of a multivariable regression analysis. In this analysis, factors that were associated with a significantly increased odds of false-negative test results compared to true-negative test results included the presence of anosmia or ageusia (aOR, 8.4; 95% CI, 1.4-50.5; *P* = .02), having had a known COVID-19–positive contact (aOR, 10.5; 95% CI, 4.3–25.4; *P* < .0001), and having an elevated LDH level (aOR, 3.3; 95% CI, 1.2–9.3; *P* = .03). A history of cancer was associated with decreased odds of false-negative test results (aOR, 0.2; 95% CI, 0.05–0.6; *P* = .008). Other variables included in the model, including race or ethnicity, other comorbidities, symptoms, and other lab and imaging findings, were not significantly associated with false-negative test results.

**Table 3.** Bivariate and Multivariable Analysis for False-Negative Test Results

Variable	Bivariate Analysis		Multivariable Analysis	
	Unadjusted OR (95% CI)	*P* Value	Adjusted OR (95% CI)	*P* Value
**Race/ethnicity**				
Black non-Hispanic	Referent			
Other non-Hispanic	2.8 (0.9–8.5)	.07	3.2 (1.0–10.5)	.06
Hispanic	0.7 (0.3–1.6)	.42	1.0 (0.4–2.4)	.98
Cancer	0.3 (0.1–0.9)	.04	0.2 (0.05–0.6)	.008
Anosmia/ageusia	7.2 (1.5–36.0)	.02	8.4 (1.4–50.5)	.02
Symptom duration >3 d	1.2 (0.6–2.3)	.59	0.9 (0.4–1.7)	.67
Known COVID-19 contacts	10.1 (4.5–22.5)	<.0001	10.5 (4.3–25.4)	<.0001
Hypoxemia	1.5 (0.8–2.9)	.20	1.3 (0.6–2.8)	.52
White blood cell count < 8.7 10^3^/µL	1.3 (0.7–2.4)	.44	1.4 (0.7–2.9)	.32
Lactate dehydrogenase > 291 U/L	3.2 (1.5–7.0)	.003	3.3 (1.2–9.3)	.03
Ferritin >356 ng/mL	2.4 (1.1–5.2)	.03	1.3 (0.5–3.5)	.64
Abnormal chest imaging	1.2 (0.6–2.2)	.60	0.8 (0.4–1.7)	.49

Note: OR, odds ratio; CI, confidence interval.

## Discussion

In this retrospective observational cohort of inpatients with possible COVID-19, we detected a false-negative testing rate of 4.0%. In multivariable analysis, the odds of false-negative testing were greater among patients presenting with anosmia or ageusia, those with known COVID-19–positive contacts, and those with elevated LDH levels. These findings suggest that clinicians should consider continuing isolation and repeated SARS-CoV-2 testing for patients who present with these clinical features.

Of all patient-reported symptoms in our study, only anosmia or ageusia was associated with increased odds of false-negative testing. The association between COVID-19 and anosmia or ageusia has been previously reported. Up to 68% of patients with COVID-19 present with some degree of olfactory dysfunction.^22^ Although olfactory dysfunction can be a feature of upper respiratory infections in general, it is more common among patients with COVID-19 compared to other pathogens and has a high specificity for COVID-19 infection.^23^

Having a known COVID-19–positive contact was also associated with false-negative testing. This finding is not surprising, given the clear risk of infection with a known exposure, but it may be less helpful for clinicians in settings where community prevalence is high and many patients report exposures.^9^ Interestingly, although nursing-home residents and healthcare workers have higher rates of infection overall, neither group had an increased risk of false-negative test results in our study.^24,25^

We also detected an association between elevated LDH levels and false-negative testing. LDH elevation is a well-described feature of COVID-19 infection and is associated with more severe illness and mortality.^18,26^ We did not find any significant association between false-negative results and other laboratory values commonly associated with COVID-19. LDH elevation may therefore be a uniquely helpful marker among patients who initially test negative, and clinicians should consider ordering it when clinical suspicion is high. However, given that we did not have LDH values for all patients in our study, this factor warrants further prospective investigation.

A history of cancer was the only clinical feature associated with decreased odds of false-negative test results in our study. This finding suggests that cancer patients with COVID-19 are more likely to be accurately identified as true positive at initial presentation. Patients with cancer are at increased risk of severe COVID-19, which may be associated with prolonged viral shedding, although data directly addressing this possibility are limited.^27,28^ Notably, we did not find this result for patients on immunosuppressive medications.

Symptom duration has been frequently cited as a reason for false-negative testing in cases in which testing is performed too early in a patient’s course. In our study, patients with false-negative test results actually had a longer median duration of symptoms (7 days vs 3 days) at the time of initial testing in bivariate analysis, although this finding was not significant in the multivariable model. A recent review by Kucirka et al^13^ reported the lowest rate of false-negative testing at 3 days after symptom onset with higher rates of false-negative testing later in the course. This finding may be explained by differences in cycle thresholds in RT-PCR testing. Recent studies have shown that cycle threshold values increase progressively from the time of symptom onset.^29^ Our laboratory does not routinely report cycle threshold data, so we were not able to include this in our analysis.

Chest CT findings have also been identified as a diagnostic clue for COVID-19 and in some cases may be even more sensitive than RT-PCR.^30^ Although both CT and chest x-ray findings have been described for COVID-19, CT seems to be a more sensitive modality, but it may not always be available in a real-world setting.^31^ In our study, abnormal chest imaging findings were more common in bivariate analysis among patients with false-negative test results, but this difference was not significant in the multivariable model. We included both CT and chest x-ray results in our study, which may have limited our ability to detect a difference given the lower sensitivity of chest x-ray for COVID-19.

Our study has several limitations. First, it was a retrospective study performed at a single center during the early peak of the pandemic among hospitalized patients with typical symptoms of influenza-like illness, so these results may not apply to other healthcare settings. Future studies should confirm these results in multiple healthcare settings in a prospective manner. Second, some of the patients classified with false-negative results may have actually been negative at the time of initial testing but acquired COVID-19 during the 7-day testing period. Although this is difficult to completely exclude, we feel that it is unlikely given that all patients in our study had symptoms consistent with COVID-19 at the time of the first test and that most secondary tests were done within 48 hours of the first test. Third, not all patients with an initial negative test were retested, so our results likely underestimate the true proportion of false-negative test results. Lastly, because diagnostic testing (including labs and imaging) was ordered at the discretion of the treating clinician, many patients had missing data which limited the robustness of our analyses.

In summary, our study provides guidance to clinicians and infection control practitioners to help make decisions surrounding use of isolation precautions and personal protective equipment for hospitalized patients with suspected COVID-19. Patients who initially test negative but who have anosmia or ageusia, known COVID-19 positive contacts, or elevated LDH levels should be strongly considered for continued isolation and repeated testing, but further studies to prospectively validate this approach are needed.
